# Data on winter daily minimum temperature and heat energy consumption in South Korea

**DOI:** 10.1016/j.dib.2020.105402

**Published:** 2020-03-16

**Authors:** Yunsoung Kim, Sanghoon Lee

**Affiliations:** a Green Energy Strategy Institute, South Korea; b New and Renewable Energy Center, Korea Energy Agency, South Korea

**Keywords:** Extreme cold, Heating energy demand, Climate change

## Abstract

This article contains the raw data on daily minimum temperature from 1976 to 2015 and energy consumption data from 1997 to 2015 in South Korea.

The daily minimum temperature data were obtained from the observed record contained in the Korea Meteorological Administration Database. The monthly heat sales data were obtained from the Korea District Heating Corporation, which is a district heating public corporation. The heating energy sales data were collected from 1997 to 2015. We considered December, February, and December as winter season.

Specifications table

**Table:** 

Subject	Energy, Atmospheric Science
Specific subject area	Extreme cold weather and heating energy demand
Type of data	Datasheets
How data were acquired	Data were collected from public database(data.kma.go.kr) and processed in Excel
Data format	Raw
Parameters for data collection	Data on dates, daily minimum temperature, and locations for extreme cold. Data on years, heating sales amount for monthly heating demand. Data on annual power peak load.
Description of data collection	Processed data from the Korea Meteorological Administration Database, Korea Power Exchange Database, and Korea District Heating Corporation
Data source location	South Korea and its weather observatories
Data accessibility	Data are provided in supplementary materials directly with this article Repository: Mendeley Data https://data.mendeley.com/datasets/v3w9yftxcg/draft?a=200f067c-a65e-4a55-8dd5-3cd39368e2fb
Related research article	Yunsoung Kim and Sanghoon lee, Trends of extreme cold events in the central regions of Korea and their influence on the heating energy demand, Weather and climate extremes, Volume 24, June 2019, Article 100,199. https://doi.org/10.1016/j.wace.2019.100199

## Value of the data

• The data provided in this article can be used for comparing the differences between the 22 observatories by the trends of the past ten years and the past 40 years.
• The data is also useful for describing the daily minimum temperature fluctuations in the 10th quantile and in the 90th quantile, so as to compare the variability in the colder quantile and warmer quantile.
• The monthly heat sales data from 1997 to 2015 are shown, which is useful to understand the variability of heating sales when the winter was colder.
• The data can be useful for the development of heating energy policies and related social supports.

## 1 Data description

The dataset provided in this article supplements the data information on the article (Kim and Lee, 2019 [1]). This article contains the raw data, provided by the Korea Meteorological Administration [2], on the daily minimum temperature on the observatories. Among the 66 observation locations, which have observation records since 1976, we exclude the stations located below latitude 35° and island area, so 44 locations are first selected.

**Fig. 1:**
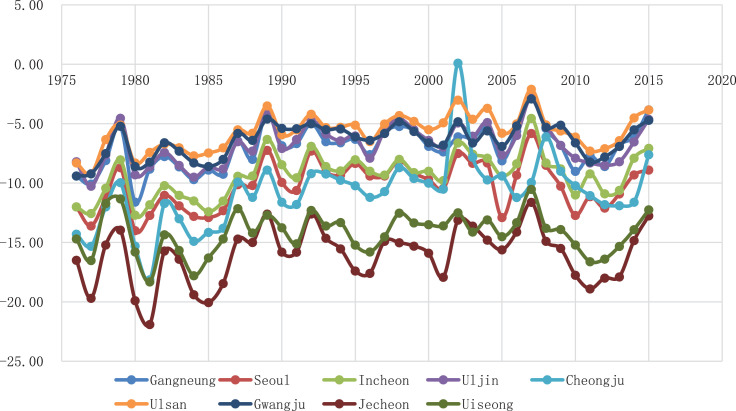
The trends of 10th quantile temperature in the 9 locations.

In order to avoid concentrating on a specific province, we excluded overly adjacent stations in the same province. As a result, about 8–9 sites in the plain or inland areas, about 6–7 sites in the mountainous area, and 7 in the coastal area are distributed in the sample. Cities with populations of more than a million are also included. Each id is an original number assigned to each station by the Korea Meteorological Administration. The 10th quantile value in each year during the 40 years in the 9 locations are as Fig. 1.

**Fig. 2:**
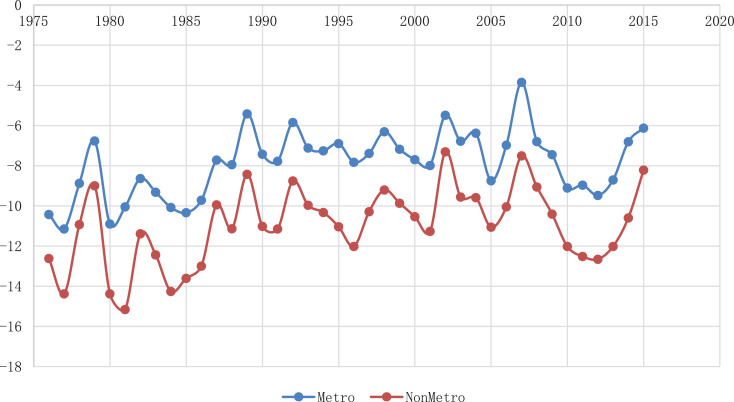
The trends of 10th quantile temperature in the metropolitan area and non-metropolitan area.

**Fig. 3:**
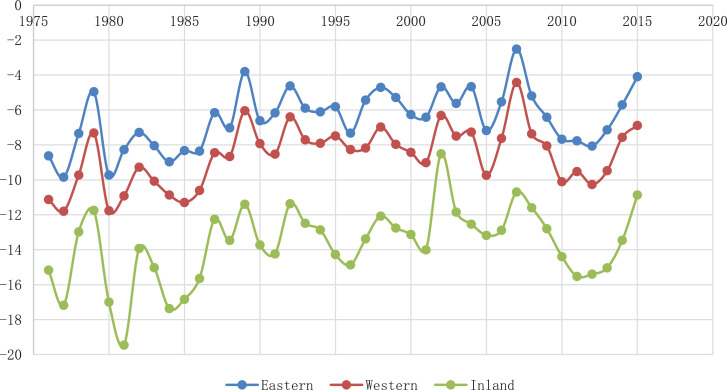
The trends of 10th quantile temperature in the eastern, western, and inland area.

The locations can be separated into two groups on the population scales, as a metropolitan area and a non-metropolitan area. Otherwise, the locations can be separated into three groups on the geographical conditions, as western area, eastern area, and inland area. In the metropolitan group, Seoul, Incheon, Gwangju, and Ulsan are included, and in the non-metropolitan group, Uljin, Cheongju, Gangneung, Jecheon, and Uiseong are included. On the geographical conditions, Seoul, Incheon, and Gwangju are the western area. Gangneung, Ulsan, and Uljin are eastern areas. Cheongju, Jecheon, and Uiseong are inland areas. The trends of each group are shown in Figs. 2 and 3.

It also provides the date of peak power demand in summer and winter. The monthly heat sales records (1997–2015) of Korea District Heating Corporation [3] can show the change in heating energy demand when there was extreme cold. District heating companies mainly sale heating energy to residential buildings, but also to commercial buildings.

## 2 Experimental design, materials, and methods

The interest of the analysis was focused on whether extreme cold is getting warmer or colder. To analyze the trends, we must define 'winter' first. If a specific temperature is a basis for determining winter, the days of winter could be various each year. We define December, January, and February as winter. After constructing the data, we performed quantile regression, which analysis method was developed by Koenker (2005 [4]). It is an advantageous method when the main research interest is on a specific quantile, rather than on average.

The quantile regression method focuses on the quantiles of the conditional distribution rather than the mean. Also, its assumptions are more flexible than ordinary least squares (OLS) regression. Generally, OLS regression supposes that the error terms are independent and identically distributed (IID), normally distributed, and homoscedastic. Quantile regression does not require these restrictive assumptions.

Extreme weather events such as heatwaves, cold waves, floods, droughts, and typhoons represent extreme values in their distribution. As extreme weather events are outliers in the data, their distribution cannot be homoscedastic. Therefore, quantile regression is a more appropriate method for the analysis of extreme weather events.

The linear quantile regression model can be written as follows (Barbosa, 2008; Koenker, 2005).

(1) $$Y_{i}=\beta_{\tau }X_{i}+u_{\tau i},\;Q_{\tau }\left(T_{i}|X_{i}\right)=\beta_{\tau }X_{i}\;\left(i-1,2,3,\ldots n\right)$$

Given a random variable *Y* with a cumulative continuous distribution function *F_Y_*(*y*), the quantile is defined as the value *Q_Y_*(*τ*) such that $P[Y\leq Q_{Y}\left(\tau \right)]=\tau$, 0< τ < 1. The quantile function *Q_Y_*(*τ*) is a reversed function of the cumulative distribution function (CDF) *F_Y_*(*y*), Therefore, $Q_{Y}\left(\tau \right)=F_{Y}^{-1}\left(\tau \right)$. Then, considering the conditional distribution of *Y* given X=x, the conditional quantile function *Q*_*Y*|*X*_(*τ; x*) verifies $P[Y\leq Q_{Y|X}\left(\tau ;x\right)|X=x]=\tau$.

β is the slope of the *τ*th quantile, and *u* is the error term. The conditional quantile function *Q* is *Y* of the *τ*th quantile. The quantile slope, *β_τ_*, is estimated from the conditional quantile function by minimizing the sum of asymmetrically weighted absolute residuals,

(2) $$Min\frac{1}{n}\left[\sum_{y_{i}\geq \beta X_{i}}\tau \left|y_{i}-\beta X_{i}\right|+\sum_{y_{i}<\beta X_{i}}\left(1-\tau \right)\left|y_{i}-\beta X{}_{i}\right|\right]$$

## Appendix Supplementary materials

Image, application 1
